# Supplementary material for: Vertical transmission of Leishmania donovani with placental degeneration in the pregnant mouse model of visceral leishmaniasis
Source: PLoS Negl Trop Dis. 2025 Jun 9;19(6):e0012650. doi: 10.1371/journal.pntd.0012650 (PMC12173239; doi:10.1371/journal.pntd.0012650)
Supplement: S1 Fig — The number of infiltrating cells in the placentas of Leishmania gene-negative and positive fetuses. The number of cells was counted in 5 random microscopic fields of placental labyrinth zone at 400 × magnification (n = 3–7). Means ± SE are presented. P values for Student’s t test are shown. (PDF) [file pntd.0012650.s001.pdf]

S1 Fig. Little relationship between placental cell infiltration and vertical transmission.

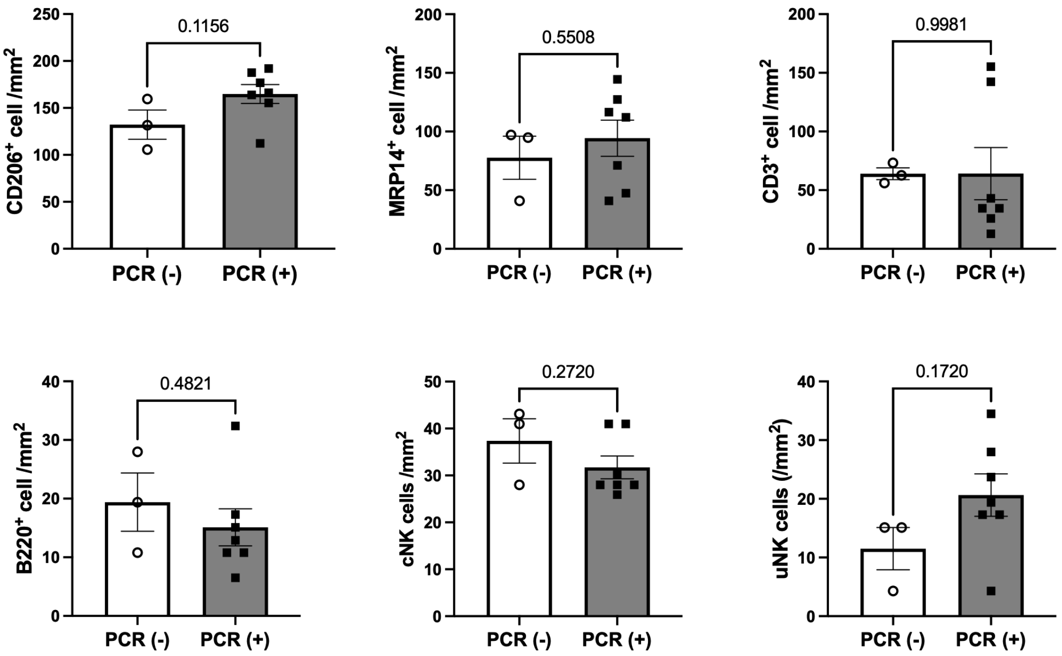

The number of infiltrating cells in the placentas of *Leishmania* gene-negative and positive fetuses. The number of cells was counted in 5 random microscopic fields of placental labyrinth zone at 400× magnification (n = 3-7). Means ± SE are presented. *P* values for Student's t test are shown.
