# Supplementary material for: Vertical transmission of Leishmania donovani with placental degeneration in the pregnant mouse model of visceral leishmaniasis
Source: PLoS Negl Trop Dis. 2025 Jun 9;19(6):e0012650. doi: 10.1371/journal.pntd.0012650 (PMC12173239; doi:10.1371/journal.pntd.0012650)
Supplement: S1 Table — (PDF) [file pntd.0012650.s002.pdf]

S1 Table. Differentially expressed genes in Ld-infected placenta.

ENSEMBL ID	gene name	base Mean	log2 FC (Ld/Naïve)	lfcSE	stat	pvalue	padj
ENSMUSG00000091694	*Apol11b*	24.0	5.23	0.67	-7.76	9.E-15	3.E-11
ENSMUSG00000073555	*Gm4951 (Iigp1c)*	61.0	2.47	0.29	-8.50	2.E-17	1.E-13
ENSMUSG00000027514	*Zbp1*	458.5	2.23	0.16	-13.92	5.E-44	8.E-40
ENSMUSG00000054072	*Iigp1*	421.3	2.19	0.39	-5.66	2.E-08	2.E-05
ENSMUSG00000032093	*Cd3e*	12.7	1.90	0.51	-3.71	2.E-04	3.E-02
ENSMUSG00000078853	*Igtp*	33.1	1.80	0.27	-6.75	1.E-11	3.E-08
ENSMUSG00000039146	*Ifi44l*	149.8	1.46	0.28	-5.19	2.E-07	1.E-04
ENSMUSG00000037321	*Tap1*	1242.4	1.38	0.15	-9.51	2.E-21	2.E-17
ENSMUSG00000067297	*Ifit1bl2*	22.1	1.34	0.37	-3.61	3.E-04	4.E-02
ENSMUSG00000068536	*Doxl2 (Aoc1l1)*	21853.1	1.33	0.36	-3.70	2.E-04	3.E-02
ENSMUSG00000020838	*Slc6a4*	199.2	1.33	0.28	-4.74	2.E-06	1.E-03
ENSMUSG00000017064	*Prl5a1*	512.2	1.29	0.35	-3.68	2.E-04	3.E-02
ENSMUSG00000055413	*H2-Q5*	126.7	1.14	0.22	-5.20	2.E-07	1.E-04
ENSMUSG00000066760	*Psg16*	45948.0	1.12	0.28	-4.03	6.E-05	1.E-02
ENSMUSG00000040264	*Gbp2b*	422.3	1.11	0.21	-5.24	2.E-07	1.E-04
ENSMUSG00000040328	*Olfr56 (Or2v1)*	68.0	1.10	0.27	-4.08	4.E-05	1.E-02
ENSMUSG00000046879	*Irgm1*	1012.9	1.10	0.19	-5.85	5.E-09	7.E-06

ENSMUSG00000023913	*Pla2g7*	2403.8	1.08	0.29	-3.75	2.E-04	3.E-02
ENSMUSG00000058153	*Sez6l*	233.2	1.08	0.27	-4.05	5.E-05	1.E-02
ENSMUSG00000073409	*H2-Q6*	1070.7	1.06	0.21	-5.11	3.E-07	2.E-04
ENSMUSG00000016496	*Cd274 (PD-L1)*	372.5	1.05	0.18	-5.87	4.E-09	6.E-06
ENSMUSG00000028270	*Gbp2*	501.2	1.05	0.20	-5.20	2.E-07	1.E-04
ENSMUSG00000021345	*Prl8a6*	39675.8	1.00	0.25	-4.05	5.E-05	1.E-02
ENSMUSG00000028268	*Gbp3*	628.1	0.99	0.21	-4.65	3.E-06	2.E-03
ENSMUSG00000060550	*H2-Q7*	1016.9	0.99	0.19	-5.09	3.E-07	2.E-04
ENSMUSG00000034459	*Ifit1*	340.2	0.98	0.21	-4.73	2.E-06	1.E-03
ENSMUSG00000069874	*Irgm2*	483.2	0.94	0.18	-5.24	2.E-07	1.E-04
ENSMUSG00000045932	*Ifit2*	367.9	0.90	0.25	-3.65	3.E-04	3.E-02
ENSMUSG00000074151	*Nlrc5*	166.8	0.88	0.16	-5.34	9.E-08	8.E-05
ENSMUSG00000049502	*Dtx3l*	569.8	0.86	0.16	-5.35	9.E-08	8.E-05
ENSMUSG00000096727	*Psmb9*	159.8	0.83	0.20	-4.09	4.E-05	1.E-02
ENSMUSG00000026104	*Stat1*	555.8	0.83	0.13	-6.42	1.E-10	2.E-07
ENSMUSG00000022906	*Parp9*	1130.7	0.82	0.13	-6.51	7.E-11	1.E-07
ENSMUSG00000068923	*Syt11*	203.7	0.82	0.22	-3.76	2.E-04	3.E-02
ENSMUSG00000040187	*Arntl2 (Bmal2)*	228.4	0.78	0.17	-4.55	5.E-06	3.E-03

ENSMUSG00000033355	*Rtp4*	680.9	0.77	0.14	-5.37	8.E-08	8.E-05
ENSMUSG00000067235	*H2-Q10*	74.0	0.76	0.19	-4.06	5.E-05	1.E-02
ENSMUSG00000030966	*Trim21*	294.1	0.76	0.13	-6.08	1.E-09	2.E-06
ENSMUSG00000060802	*B2m*	10218.6	0.75	0.17	-4.32	2.E-05	5.E-03
ENSMUSG00000058729	*Lin9*	126.1	0.73	0.16	-4.45	9.E-06	3.E-03
ENSMUSG00000055994	*Nod2*	135.7	0.72	0.18	-4.09	4.E-05	1.E-02
ENSMUSG00000029561	*Oasl2*	1849.1	0.72	0.15	-4.72	2.E-06	1.E-03
ENSMUSG00000091705	*H2-Q2*	246.9	0.69	0.17	-3.97	7.E-05	1.E-02
ENSMUSG00000056116	*H2-T22*	1006.7	0.69	0.15	-4.66	3.E-06	2.E-03
ENSMUSG00000073411	*H2-D1*	14082.3	0.67	0.16	-4.29	2.E-05	5.E-03
ENSMUSG00000019487	*Trip10*	1083.7	0.67	0.16	-4.17	3.E-05	8.E-03
ENSMUSG00000067203	*H2-K2*	157.8	0.67	0.17	-3.90	1.E-04	2.E-02
ENSMUSG00000040033	*Stat2*	1706.9	0.66	0.10	-6.76	1.E-11	3.E-08
ENSMUSG00000032596	*Uba7*	508.1	0.65	0.12	-5.38	7.E-08	8.E-05
ENSMUSG00000073402	*Gm8909 (H2-T26)*	240.2	0.63	0.14	-4.36	1.E-05	5.E-03
ENSMUSG00000018899	*Irf1*	881.3	0.63	0.16	-3.98	7.E-05	1.E-02
ENSMUSG00000073403	*Gm10499 (H2-T7)*	734.3	0.62	0.15	-4.01	6.E-05	1.E-02
ENSMUSG00000063268	*Parp10*	933.1	0.61	0.08	-7.77	8.E-15	3.E-11

ENSMUSG00000079507	*H2-Q1*	987.1	0.60	0.15	-4.16	3.E-05	9.E-03
ENSMUSG00000035929	*H2-Q4*	2785.3	0.60	0.13	-4.52	6.E-06	3.E-03
ENSMUSG00000027639	*Samhd1*	2125.9	0.58	0.12	-4.97	7.E-07	4.E-04
ENSMUSG00000029605	*Oas1b*	123.5	0.58	0.14	-4.17	3.E-05	9.E-03
ENSMUSG00000061232	*H2-K1*	3237.6	0.58	0.14	-4.02	6.E-05	1.E-02
ENSMUSG00000018770	*Atp5g3*	2049.8	-0.37	0.09	4.30	2.E-05	5.E-03
ENSMUSG00000006057	*Atp5g1*	689.2	-0.39	0.11	3.66	2.E-04	3.E-02
ENSMUSG00000033938	*Ndufb7*	894.4	-0.42	0.12	3.60	3.E-04	4.E-02
ENSMUSG00000029632	*Ndufa4*	1556.6	-0.46	0.11	4.06	5.E-05	1.E-02
ENSMUSG00000016252	*Atp5e*	430.4	-0.46	0.11	4.30	2.E-05	5.E-03
ENSMUSG00000025651	*Uqcrc1*	2855.5	-0.47	0.13	3.54	4.E-04	5.E-02
ENSMUSG00000026202	*Tuba4a*	1277.1	-0.61	0.14	4.34	1.E-05	5.E-03
ENSMUSG00000001985	*Grik3*	437.7	-0.63	0.14	4.59	4.E-06	2.E-03
ENSMUSG00000007039	*Ddah2*	1898.6	-0.64	0.16	4.10	4.E-05	1.E-02
ENSMUSG00000015441	*Gzmf*	2159.7	-0.65	0.17	3.86	1.E-04	2.E-02
ENSMUSG00000040284	*Gzmg*	2852.6	-0.68	0.19	3.63	3.E-04	4.E-02
ENSMUSG00000006310	*Zbtb32*	92.0	-0.69	0.20	3.53	4.E-04	5.E-02
ENSMUSG00000040424	*Hipk4*	114.2	-0.70	0.19	3.79	2.E-04	2.E-02

ENSMUSG00000015437	*Gzmb*	207.3	-0.71	0.20	3.65	3.E-04	3.E-02
ENSMUSG00000059256	*Gzmd*	3072.8	-0.73	0.19	3.80	1.E-04	2.E-02
ENSMUSG00000000489	*Pdgfb*	2298.9	-0.76	0.21	3.56	4.E-04	4.E-02
ENSMUSG00000038296	*Galnt18*	609.7	-0.77	0.17	4.59	5.E-06	2.E-03
ENSMUSG00000037904	*Ankrd9*	312.5	-0.78	0.19	4.10	4.E-05	1.E-02
ENSMUSG00000079186	*Gzmc*	1043.7	-0.79	0.19	4.08	5.E-05	1.E-02
ENSMUSG00000037202	*Prf1*	442.0	-0.82	0.19	4.25	2.E-05	6.E-03
ENSMUSG00000070504	*Fcrl6*	124.0	-0.83	0.24	3.54	4.E-04	5.E-02
ENSMUSG00000022156	*Gzme*	1697.2	-0.85	0.20	4.26	2.E-05	6.E-03
ENSMUSG00000040314	*Ctsg*	74.2	-0.86	0.22	3.93	8.E-05	2.E-02
ENSMUSG00000048782	*Insc*	382.0	-0.88	0.22	3.99	7.E-05	1.E-02
ENSMUSG00000035964	*Tmem59l*	190.0	-0.91	0.20	4.52	6.E-06	3.E-03
ENSMUSG00000037493	*Cib2*	108.3	-1.01	0.18	5.69	1.E-08	2.E-05
ENSMUSG00000015981	*Stk32c*	67.8	-1.33	0.30	4.37	1.E-05	5.E-03
ENSMUSG00000050370	*Ch25h*	24.2	-1.42	0.40	3.58	3.E-04	4.E-02
ENSMUSG00000048473	*Sult6b2*	25.2	-1.56	0.44	3.52	4.E-04	5.E-02
ENSMUSG00000038204	*Asb10*	24.7	-1.71	0.48	3.58	3.E-04	4.E-02
