# Supplementary material for: Vertical transmission of Leishmania donovani with placental degeneration in the pregnant mouse model of visceral leishmaniasis
Source: PLoS Negl Trop Dis. 2025 Jun 9;19(6):e0012650. doi: 10.1371/journal.pntd.0012650 (PMC12173239; doi:10.1371/journal.pntd.0012650)
Supplement: S2 Table — (PDF) [file pntd.0012650.s003.pdf]

S2 Table. Genes related to vasodilation in Ld-infected placenta.

ENSEMBL ID	gene name	base Mean	log2 FC (Ld/Naïve)	lfcSE	stat	pvalue	padj
ENSMUSG00000020826	*Nos2*	2711.0	-0.08	0.15	0.51	6.E-01	9.E-01
ENSMUSG00000026820	*Ptges2*	444.7	-0.36	0.14	2.54	1.E-02	2.E-01
ENSMUSG00000023951	*Vegfa*	6260.2	0.08	0.12	-0.66	5.E-01	8.E-01
ENSMUSG00000024962	*Vegfb*	245.2	0.18	0.16	-1.12	3.E-01	7.E-01
ENSMUSG00000031520	*Vegfc*	43.2	0.13	0.29	-0.45	6.E-01	9.E-01
