# Supplementary material for: Vertical transmission of Leishmania donovani with placental degeneration in the pregnant mouse model of visceral leishmaniasis
Source: PLoS Negl Trop Dis. 2025 Jun 9;19(6):e0012650. doi: 10.1371/journal.pntd.0012650 (PMC12173239; doi:10.1371/journal.pntd.0012650)
Supplement: S3 Table — (PDF) [file pntd.0012650.s004.pdf]

S3 Table. IFN genes in Ld-infected placenta, spleen and liver.

ENSEMBL ID	gene name	base Mean	log2 FC (Ld/Naïve)	lfcSE	stat	pvalue	padj
**Placenta**							
ENSMUSG00000095498	*Ifna1*	0	NA	NA	NA	NA	NA
ENSMUSG00000078354	*Ifna2*	0	NA	NA	NA	NA	NA
ENSMUSG00000048806	*Ifnb1*	0.2	1.03	3.16	-0.33	7.E-01	NA
ENSMUSG00000045364	*Ifne*	8.3	-0.41	0.82	0.50	6.E-01	NA
ENSMUSG00000042993	*Ifnk*	81.4	0.49	0.40	-1.23	2.E-01	6.E-01
ENSMUSG00000055170	*Ifng*	0.4	-1.23	2.51	0.49	6.E-01	NA
**Spleen**							
ENSMUSG00000095498	*Ifna1*	0.1	-0.41	3.16	-0.13	9.E-01	NA
ENSMUSG00000078354	*Ifna2*	0.1	0.75	3.16	0.24	8.E-01	NA
ENSMUSG00000048806	*Ifnb1*	0.3	-1.75	3.12	-0.56	6.E-01	NA
ENSMUSG00000045364	*Ifne*	0.0	NA	NA	NA	NA	NA
ENSMUSG00000042993	*Ifnk*	0.1	-0.41	3.16	-0.13	9.E-01	NA
ENSMUSG00000055170	*Ifng*	256.8	3.31	0.37	9.06	1.E-19	7.E-18
**Liver**							
ENSMUSG00000095498	*Ifna1*	0	NA	NA	NA	NA	NA
ENSMUSG00000078354	*Ifna2*	0	NA	NA	NA	NA	NA
ENSMUSG00000048806	*Ifnb1*	0	NA	NA	NA	NA	NA
ENSMUSG00000045364	*Ifne*	0	NA	NA	NA	NA	NA
ENSMUSG00000042993	*Ifnk*	0	NA	NA	NA	NA	NA
ENSMUSG00000055170	*Ifng*	50.0	6.04	0.63	9.58	9.5E-22	4.8E-20

NA: Not available
