# Supplementary material for: Vertical transmission of Leishmania donovani with placental degeneration in the pregnant mouse model of visceral leishmaniasis
Source: PLoS Negl Trop Dis. 2025 Jun 9;19(6):e0012650. doi: 10.1371/journal.pntd.0012650 (PMC12173239; doi:10.1371/journal.pntd.0012650)
Supplement: Attachment — Submitted filename: Response to Reviewers 250512.docx [file pntd.0012650.s005.docx]

**Response to Reviewers**

We appreciate very much the reviewer’s suggestions and believe that the constructive comments are valuable for improving our manuscript. Revisions to the manuscript that were performed according to the reviewer’s comments are shown in red.

**Reviewer comments:**

**Methods**

Q1. **Fig. 4B. It is not clear how may mice/organs/sections were evaluated to conclude that KMP11^+^ cells are detectable in fetal liver, but ‘only a few KMP11^+^ cells were observed in the placentas’, especially when Fig. 4B only shows a single positive cell in the image. Quantification of microscopy would strengthen the observation of this novel finding.**
A1. KMP11^+^ cells were assessed in fetal liver (n=11) and placenta (n=21) of Ld-infected mice. In the fetal livers of Ld-infected group, KMP11^+^ cells were detected in 4 of 11 mice, with the cell numbers ranging from 1 to 2 per section. In the placentas of Ld-infected group, KMP11^+^ cells were detected in 3 of 21 mice, with cell numbers ranging from 1 to 3 per section.

In response to the reviewer’s suggestion, we have added the following sentences to Result section (p. 16, line 306):

(p. 16, line 306)
Based on these results, we performed immunostaining for the *Leishmania* antigen KMP11, and KMP11^+^ cells were detected in the fetal liver (Fig 4B). KMP11^+^ cells were detected in the fetal livers of Ld-infected mice (4/11 mice), with the cell numbers ranging from 1 to 2 per section. This is the first report to histologically demonstrate Ld vertical transmission in a VL mouse model. On the other hand, KMP11^+^ cells were infrequently detected in the placentas of Ld-infected mice, observed in 3 out of 21 samples with 1 to 3 cells per section. The result demonstrated vertical transmission in our VL mouse model.

Q2. **Line 307: Quantification is needed to support ‘the density of the LZ was lower than that of naïve controls.**
A2. Following the reviewer's suggestion, the tissue area of the labyrinth zone was quantified, and the data was added to Fig 4C. The area of the labyrinth zone was quantified by specifying and measuring the tissue area of the labyrinth zone excluding the vascular lumen using an all-in-one fluorescence microscope BZ-X800 (KEYENCE, Osaka, Japan) and its analyzer.

    Correspondingly, the following sentences were added to the Materials and methods section (p. 11, line 207), and the figure legend for Fig 4C (p. 54, line 1046) was updated:

    (p. 11, line 207)
    The labyrinth zone tissue area excluding the vascular lumen was measured using an all-in-one fluorescence microscope BZ-X800 and its analyzer (KEYENCE, Osaka, Japan) at 200× magnification.

    (p. 54, line 1045)
    Fig 4. (C) Left: Ld-induced placental degeneration. Trophoblastic cell atrophy (arrow) and vasodilation (arrowhead) were observed in HE-stained infected placentas. Bars, 20 μm. Right: Labyrinth zone tissue area excluding vascular lumen at 200× magnification (n=5-21). Means ± SE are presented. *P* value for Student’s t test is shown.
Q3. **Was IFNa/b or IFNg increased in RNASeq data? IFNg is decreased with pregnancy in infected mice, but it is still elevated compared to naïve/pregnancy or infected/non pregnant. Similarly, were type I IFNs elevated in the serum like IFNg in Fig. 2B?**
A3. Although we did not measure serum levels of type I IFN in this study, bulk RNA-seq analysis of the placenta, spleen, and liver from Ld-infected mice showed no upregulation of type I IFN expression (see S3Table). Similarly, type I IFNs have not been detected in the serum of human VL patients (S. Sukla et al., 2022, Sci Rep, 12(1):14436). However, RNA-seq analysis of peripheral blood leukocytes from VL patients has shown activation of type I IFN signaling, suggesting a potential involvement in disease progression (R. Kumar et al., Cell Rep, 2020, 30(8): 2512–2525.e9).

These findings indicate that even when type I IFN levels are low in serum or whole tissues, IFN signaling can be activated locally in specific sites, and the placenta is thought to be one such example. Indeed, the placenta may be a unique immune environment in which IFN signaling is more likely to be activated (J. Ding et al., 2022, Immunol Rev, 308(1): 9–24).

Furthermore, it is known that interactions with toll-like receptors and the STING pathway can enhance IFN signaling (A. Bowie, 2012, Sci Signal, 5(214):pe9; B. Temizoz et al., 2015, Eur J Immunol, 45(4):1159–69), which may also contribute to the localized activation. In addition, an IFN-independent ISG induction pathway has also been reported in leishmaniasis (M. Rossi et al., 2017, PNAS, 114(19):4987–4992).

Taken together, these findings suggest that even in the absence of detectable type I IFN in serum, local activation of IFN signaling in the placenta is entirely plausible. This may be attributed to the placenta's unique immune environment and the involvement of diverse signaling pathways.

In response to the reviewer’s suggestion, we have added the following sentences and S3 Table to Discussion section (p. 29, line 519):

(p. 29, line 519)
Although we did not measure serum levels of type I IFN in this study, bulk RNA-seq analysis of the placenta, spleen, and liver from Ld-infected mice showed no upregulation of type I IFN expression (S3Table). Similarly, type I IFNs have not been detected in the serum of human VL patients [110]. However, RNA-seq analysis of peripheral blood leukocytes from VL patients has shown activation of type I IFN signaling, suggesting a potential involvement in disease progression [111]. These findings indicate that even when type I IFN levels are low in serum or whole tissues, IFN signaling can be activated locally in specific sites, and the placenta is thought to be one such example. Indeed, the placenta is a unique immune environment in which IFN signaling is more likely to be activated [112]. Furthermore, it is known that interactions with toll-like receptors and the STING pathway can enhance IFN signaling [113,114], which may also contribute to the localized activation. In addition, an IFN-independent ISG induction pathway has also been reported in leishmaniasis [108]. Taken together, these findings suggest that even in the absence of detectable type I IFN in serum, local activation of IFN signaling in the placenta is entirely plausible. This may be attributed to the placenta's unique immune environment and the involvement of diverse signaling pathways.

Q4. **Fig. 5C, lines 324-325. The data does not support more uNK cells, so the emphasis should be decreased on their infiltration in line 325, 467, and 473.**
A4. As the reviewer pointed out, we have deleted the sentence referring to the increase in uNK cells in accordance with the data (p. 18, line 336; p. 28, line 498; p. 54, line 1051).
Q5. **Describe how estimate Ld number in fetus was calculated in M&M (Fig. 4A).**
A5. I apologize for not describing the method. Estimates of fetal Ld numbers were calculated using quantitative PCR by fitting the Ct values of DNA samples extracted from whole fetal livers to a standard curve constructed from known numbers of cultured Ld parasites.

    In response to the reviewer’s comment, we have added the following sentences to Material and methods section (p. 9, line 178):

    (p. 9, line 178)
    Estimates of fetal Ld numbers were calculated using quantitative PCR by fitting the Ct values of DNA samples extracted from whole fetal livers to a standard curve constructed from known numbers of cultured Ld parasites.

**Discussions**

Q1. **Discussion: What causes the vessel diameter? Are vascular mediators like VEGF-A elevated in the RNASeq dataset that couple help explain the dilation?**
A1. As pointed out by the reviewer, there are several factors that could contribute to vascular dilation in the placenta, including VEGF, as outlined below:
1) Nitric oxide (NO)
2) Prostaglandin E2 (PGE2)
3) Vascular endothelial growth factor (VEGF)
4) Hypoxic conditions

Regarding factors 1 to 3, RNA-seq data from Ld-infected placentas showed no changes in the *Nos2*, *Ptges2*, or VEGF genes (S2 Table). On the other hand, in Ld-infected placentas, genes related to oxidative phosphorylation, including *Atp5g3*, *Atp5g1*, *Ndufb7*, *Ndufa4*, *Atp5e*, and *Uqcrc1*, were downregulated, while the *Arntl2* gene, which is induced in response to hypoxia [98], was upregulated (S1 Table). Consistent with this, it has been reported that hypoxic conditions are induced during Ld infection [99]. These results suggest that the hypoxic environment induced by Ld infection in the placenta may contribute to vascular dilation.

In response to the reviewer’s suggestion, we have added the following sentences and S2 Table to Discussion section (p. 27, line 481):

(p. 27, line 481)
Possible factors of vascular dilation in the placenta include nitric oxide (NO), prostaglandin E2 (PGE2), vascular endothelial growth factor (VEGF), and hypoxic conditions. RNA-seq data from Ld-infected placentas showed no significant changes in the *Nos2*, *Ptges2*, and VEGF genes (S2 Table). On the other hand, in Ld-infected placentas, genes related to oxidative phosphorylation, including *Atp5g3*, *Atp5g1*, *Ndufb7*, *Ndufa4*, *Atp5e*, and *Uqcrc1*, were downregulated, while the *Arntl2* gene, which is induced in response to hypoxia [98], was upregulated (S1 Table). Consistent with this, it has been reported that hypoxic conditions are induced during Ld infection [99]. These results suggest that the hypoxic environment induced by Ld infection in the placenta may contribute to vascular dilation.

Q2. **Discussion: Is elevated ICAM and VCAM due to elevated IFNg?**
A2. As pointed out by the reviewer, it has been reported that IFNγ induces the expression of ICAM1 and VCAM1 (RD Caterina et al., 2001, Arterioscler Thromb Vasc Biol. 21(2):227-32; YJ Chang et al., 2002, J Biol Chem.277(9):7118-26). Therefore, the increase in IFNγ due to Ld infection may be involved in inducing ICAM1 and VCAM1 expression in the placenta.

    In response to the reviewer’s suggestion, we have added the following sentences to Discussion section (p. 27, line 490):

(p. 27, line 490)
In addition, villitis accompanied by T cell infiltration was observed, and the affected areas corresponded with the localization of ICAM1 and VCAM1 (Fig 5). Since IFN-γ has been reported to induce the expression of ICAM1 and VCAM1 [100,101], it is possible that the increased levels of IFN-γ during Ld infection contribute to the upregulation of ICAM1 and VCAM1 expression in the placenta.

Q3. **What restricts the CD3 T cells from moving throughout the tissue or why or the cells cluster?**
A3. The reason is still unclear, but the pathology of Ld-infected placentas is similar to that of granulomatous chronic villitis (PJ Katzman et al., 2011, Pediatr Dev Pathol.14(4):284-93). In VL livers, granulomas centered around infected macrophages and parasite antigens are observed. Therefore, it is likely that, similar to the VL livers, a granuloma-like pathology could also be observed in the placenta.
Q4. **Line 335-336: no control group is mentioned. Placentas of Ld infected compared to...**
A4. Apologies for the lack of detail. We have added a description regarding the control group (p18, line 347).
Q5. **Is BALA/c a typo?**
A5. As the reviewer pointed out, this was a typographical error. Apologies for the oversight. It has been corrected to BALB/cA (p26, line 462).
Q6. **Line 451 need a citation.**
A6. Although the citation was included, as the reviewer rightly pointed out, the formatting was unclear. We have therefore moved the citation to the end of the sentence for clarity (p26, line 465).

    (p26, line 465)

Before revision:

Additionally, *L. panamensis* [28] and *L. mexicana* [94], which cause cutaneous leishmaniasis, have also been reported to cause vertical transmission in hamsters and mice.

After revision:

Additionally, *L. panamensis* and *L. mexicana*, which cause cutaneous leishmaniasis, have also been reported to cause vertical transmission in hamsters and mice [28,94].

**Conclusions**

Q1. **Many genes mentioned in Table 3 are induced by either type I IFNs or IFNg, using STAT1 as a prime example. Therefore, the conclusion that type I IFNs are solely responsible is not obvious or supported without pathway analysis or uncoupling the roles of each pathway through blockade strategies.**
A1. As the reviewer pointed out, many of the genes upregulated in Ld-infected placentas in our data are induced by either type I IFN or IFN-γ. Given that IFN-γ levels increase during Ld infection, it is difficult to attribute these changes solely to the effects of type I IFN; the potential involvement of IFN-γ in the pathology should also be considered. In fact, while IFN-γ is a key mediator of the Th1 immune response, excessive activation of this cytokine has been reported to contribute to immune-mediated organ damage and chronic inflammation in visceral leishmaniasis (P Kaye et al., 2011, Nat Rev Microbiol, 9(8):604–15). Moreover, high levels of IFN-γ have also been implicated in placentitis and impaired placental development (L Yockey et al., 2018, Immunity, 49(3):397–412). Therefore, both type I IFNs and IFN-γ may play complex roles in the development of placental inflammation in pregnant VL, and further experimental studies are needed to clarify their respective contributions.

Based on the above, we have added references (ref. 119, 121, 130) and replaced “type I IFN” with “IFN” in the relevant sections (p. 2, lines 35–37; p. 3, line 47; p. 28, lines 511–518; p. 30, lines 542–569). In addition, we have added the following sentences to the Discussion section (p. 29, line 533):

(p. 29, line 533)
It is important to note that many of the genes upregulated in the Ld-infected placenta may be induced not only by type I IFN but also by IFN-γ. Furthermore, considering that IFN-γ levels increase in the serum during Ld infection (Fig 2B), it is difficult to conclude that these changes are solely due to type I IFN. The potential involvement of IFN-γ in the disease pathology must also be taken into account. Although IFN-γ is a central mediator of the Th1 immune response, its excessive activation has been associated with immune-mediated organ damage and chronic inflammation in VL [134]. Therefore, in pregnant VL, both type I IFN and IFN-γ may be intricately involved in the pathogenesis of placental inflammation, and further experimental studies are needed to clarify their respective roles.

Q2. **To make the conclusion that barrier integrity is affected, the authors would need to assess barrier integrity using a common assay like evans blue which measures leakiness, or a comparable approach.**
A2. As pointed out by the reviewer, there were some instances where the expression was overly strong. Therefore, we have revised the relevant sections. Specifically, we have removed the phrase "resulting in disruption of the placental barrier" from the conclusion (p. 31, line 567):

(p. 31, line 567)
Before revision:

The results suggest that IFN induces cellular immunosuppression (providing an environment favorable for protozoan survival) and placental degeneration (resulting in disruption of the placental barrier), which contribute to vertical transmission.

After revision:

The results suggest that IFN induces cellular immunosuppression (providing an environment favorable for protozoan survival) and placental degeneration, which contribute to vertical transmission.

**Editorial and Data Presentation Modifications**

Q1. **Arrows denoting LZ, trophoblast atrophy, and vasodilation would help the reader for Fig. 4C. For instance, it should be made clear if the nuclei are of villi or trophoblasts?**
A1. In response to the reviewer’s comment, we have added arrows indicating trophoblast cell atrophy and arrowheads indicating vascular dilation in the HE image of the placenta in Fig. 4C. Additionally, we have included corresponding details in the figure legend (p. 54, line 1045).

    The labyrinth zone of the mouse placenta has a spongy structure, with maternal blood spaces and fetal blood vessels interwoven, and two layers of syncytiotrophoblasts surrounding the blood vessels. However, it is important to note that in the HE-stained image, the boundaries are unclear, making it difficult to distinguish the villi as clearly as in human placenta.

**Summary and General Comments**

Q1. **This study shows that chronic Ld infection induces decreased pregnancy rate, vertical transmission, placental degeneration with T cell infiltration. The manuscript is well-written and addresses an important unanswered question in NTDs. Plus, the new experimental model developed will be informative for future studies dissecting the immunopathology associated with pregnancy in VL. However, the conclusion that type I IFN signaling in the placentas is responsible for the phenotype is less convincing as many of the targets identified by RNASeq can be induced by type I IFNs and IFNg. Therefore, the conclusion that type IFNs are important should be dampened (in the results, discussion, etc) as the current study does not uncouple IFNa/b from IFNg.**
A3. As this point has already been addressed above, please kindly excuse any repetition in my response.

    As the reviewer pointed out, many of the genes upregulated in Ld-infected placentas in our data are induced by either type I IFN or IFN-γ. Given that IFN-γ levels increase during Ld infection, it is difficult to attribute these changes solely to the effects of type I IFN; the potential involvement of IFN-γ in the pathology should also be considered. In fact, while IFN-γ is a key mediator of the Th1 immune response, excessive activation of this cytokine has been reported to contribute to immune-mediated organ damage and chronic inflammation in visceral leishmaniasis (P Kaye et al., 2011, Nat Rev Microbiol, 9(8):604–15). Moreover, high levels of IFN-γ have also been implicated in placentitis and impaired placental development (L Yockey et al., 2018, Immunity, 49(3):397–412). Therefore, both type I IFNs and IFN-γ may play complex roles in the development of placental inflammation in pregnant VL, and further experimental studies are needed to clarify their respective contributions.

Based on the above, we have added references (ref. 119, 121, 130) and replaced “type I IFN” with “IFN” in the relevant sections (p. 2, lines 35–37; p. 3, line 47; p. 28, lines 511–518; p. 30, lines 542–569). In addition, we have added the following sentences to the Discussion section (p. 29, line 533):

(p. 29, line 533)
It is important to note that many of the genes upregulated in the Ld-infected placenta may be induced not only by type I IFN but also by IFN-γ. Furthermore, considering that IFN-γ levels increase in the serum during Ld infection (Fig 2B), it is difficult to conclude that these changes are solely due to type I IFN. The potential involvement of IFN-γ in the disease pathology must also be taken into account. Although IFN-γ is a central mediator of the Th1 immune response, its excessive activation has been associated with immune-mediated organ damage and chronic inflammation in VL [134]. Therefore, in pregnant VL, both type I IFN and IFN-γ may be intricately involved in the pathogenesis of placental inflammation, and further experimental studies are needed to clarify their respective roles.
